# Left ventricular and left atrial volume ratio assessed by three‐dimensional echocardiography: Novel indices for evaluating age‐related change in left heart chamber size

**DOI:** 10.14814/phy2.14300

**Published:** 2019-12-09

**Authors:** Masaaki Takeuchi, Tetsuji Kitano, Yosuke Nabeshima, Yutaka Otsuji, Kyoko Otani

**Affiliations:** ^1^ Department of Laboratory and Transfusion Medicine School of Medicine Hospital of University of Occupational and Environmental Health Kitakyushu Japan; ^2^ Second Department of Internal Medicine School of Medicine University of Occupational and Environmental Health Kitakyushu Japan

**Keywords:** 3D echocardiography, left ventricular‐left atrial volume ratio, reference values

## Abstract

We hypothesized that left ventricular and left atrial volume ratio (LVLAVR) assessed by three‐dimensional echocardiography (3DE) reflects age‐ and gender‐related change in left chamber size. We aimed to (1) determine the reference values of LVLAVR, (2) investigate their age and gender dependency, and (3) clarify which anthropometric and echocardiography parameters are closely associated with these indices. Both left ventricular (LV) and left atrial (LA) volume curves were obtained using 3DE speckle tracking analytical software, and the LVLAVR curve throughout one cardiac cycle was created, from which LVLAVR at ventricular end‐diastole and at ventricular end‐systole were determined in 313 healthy subjects (age, 20–85 years; 51% men). The mean values of LVLAVR at ventricular end‐diastole and ventricular end‐systole in male subjects were 5.74 ± 1.54 and 1.37 ± 0.35, respectively. Corresponding values in female subjects were significantly lower (5.20 ± 1.47, *p* = .003 and 1.13 ± 0.29, *p* < .001) than the values in male subjects. LVLAVR at ventricular end‐diastole step wisely decreased to advanced aging, and had a highest F ratio compared with other left chamber volumetric parameters in both genders. LV mass and LA ejection fraction were significantly associated with LVLAVR at ventricular end‐diastole. In contrast, LV mass and LV ejection fraction were significantly coupled with LVLAVR at ventricular end‐systole. This study provides the reference values for LVLAVR from a relatively large number of healthy subjects. LVLAVR may be a sensitive parameter to reflect age‐ and gender‐related change in LV and LA volumes. Further studies should be required to determine its clinical usefulness over traditional echocardiography parameters in various cardiovascular diseases.

## Graphical abstract

To our best knowledge, this study is the first to investigate the left ventricular and left atrial volume ratio (LVLAVR) using three‐dimensional echocardiography. We determined age‐ and sex‐specific reference values of LVLAVR at end‐diastole and end‐systole from a relatively large number of healthy subjects. Detailed assessment of LVLAVR clarifies the temporal status of LV−LA coupling at different time points of the cardiac cycle, and thus, would provide further insights for LV−LA coupling.

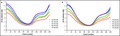

## 1 INTRODUCTION

Age‐ and gender‐related change in left ventricular (LV) and left atrial (LA) volumes has been reported in healthy subjects (Badano et al., 2016; Kaku et al., 2011). The left ventricle and left atrium are contiguous left heart chambers, and become a conduit during diastole. Thus, it is intuitively recognized that both chambers affect each other during different phases of the cardiac cycle. Although evaluation of LV and LA volumes using two‐dimensional echocardiography (2DE) is a fundamental approach in echocardiography laboratories (Lang et al., 2015), their measurements may not be accurate due to geometric assumptions. Three‐dimensional echocardiography (3DE) can potentially provide accurate and reliable measurements of LV and LA volumes. Several studies have determined the reference values of LV volumes and LV ejection fraction (LVEF) (Aune, Baekkevar, Rodevand, & Otterstad, 2010; Bernard et al., 2017; Chahal et al., 2012; Fukuda et al., 2012; Kaku et al., 2011; Muraru et al., 2013). Although some studies have addressed normal values of 3DE determined LA volumes, majority of current 3DE quantification software was not aimed for LA volume analysis, or it required extensive and tedious manual tracing on LA endocardial border at several time points of the cardiac cycle (Aune, Bækkevar, Roislien, Rodevand, & Otterstad, 2009; Badano et al., 2016; Russo et al., 2017; Wu et al., 2013). Recently, a novel semiautomatic 3DE software, which has capability of both LV and LA volume measurements throughout one cardiac cycle, has been developed.

We hypothesized that LV and LA volume ratio (LVLAVR) is a sensitive parameter to detect age‐ and gender‐related change in left chamber size. We also hypothesized that this ratio would reflect the temporal status of LV–LA coupling at different phases of the cardiac cycle, and the contribution of other anthropometric and echocardiography parameters for LVLAVR is different at different time points of the cardiac cycle.

Accordingly, the aims of this study were to (1) determine the reference values of LVLAVR in healthy subjects, (2) investigate their age and sex dependency, and (3) clarify which anthropometric and echocardiography parameters are closely associated with these indices.

## 2 METHODS

### 2.1 Study population

Using 3DE database, we retrospectively searched adult healthy Japanese volunteers without hypertension, diabetes, hypercholesterolemia, and cardiovascular disease who underwent 3DE examination. Among 313 subjects who fulfilled eligibility criteria, majority were hospital employees and their relatives, residents, and fellows. Some 3DE datasets used for this study have been used for the previous publications (Kaku et al., 2011, 2014; Mizukoshi et al., 2016; Wu et al., 2013). The Ethics Committee of the University of Occupational and Environmental Health approved the study protocol; the need for obtaining informed consent was waived due to the retrospective nature of the analysis.

### 2.2 Echocardiographic acquisition

All subjects underwent a comprehensive 2DE and Doppler echocardiography study. LV inflow velocity was obtained at the level of the mitral leaflet tips. Mitral annular velocity on tissue Doppler echocardiography was recorded at both sides of the mitral annulus. Full‐volume 3DE datasets’ acquisition was performed by apical approach using commercially available ultrasound machine and equipment (iE33 with X3‐1 or X 5‐1 probe or Epic 7G with X5‐1 probe, Philips Healthcare, Andover, MA; E95 with 4V probe, GE Healthcare, Horten, Norway). The depth and sector angle were adjusted to include the entire left ventricle and left atrium with the highest frame rate. 3DE full‐volume datasets were acquired with multibeat acquisition.

### 2.3 3DE analysis

**Figure 1:**
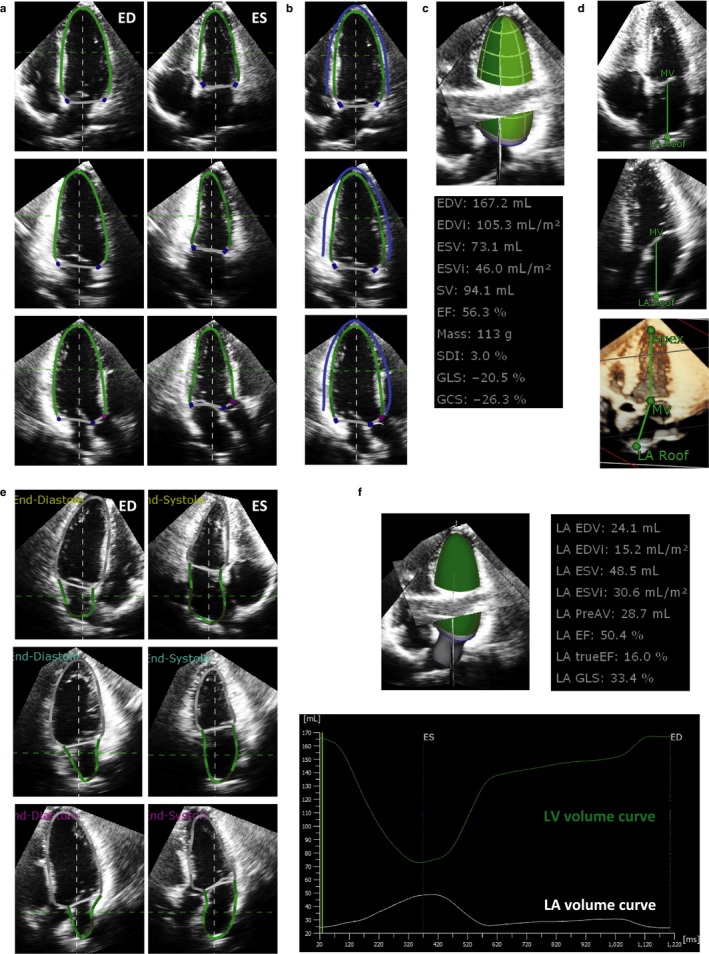
Step‐by‐step approach of left ventricular and left atrial volume measurements. (a) Left ventricular (LV) endocardial border tracing (green lines) on apical four‐chamber (upper panels), two‐chamber (middle panels), and long‐axis (lower panels) views at end‐diastole (ED) and end‐systole (ES). (b) LV mass calculation. Blue contour represents LV epicardial casts, from which LV mass was determined as (LV epicardial cast volume − LV endocardial cast volume) × 1.05. (c) Achievement of LV geometrical and functional assessment. (d) Initialization of left atrial (LA) endocardial contour. The center of the mitral valve and LA roof was determined on apical four‐chamber and long‐axis views. Note that the LV long axis and LA long axis lines are not parallel but angulated. (e) LA border determination on apical four‐chamber (upper panels), two‐chamber (middle panels), and long‐axis (lower panels) views at end‐diastole (ED) and end‐systole (ES). (f) Results of LA geometrical and functional assessment. LV and LA volume curves during one cardiac cycle are also shown

The image quality of 3DE datasets were evaluated subjectively, considering the completeness of LV and LA border visualization, and categorized as good, fair, poor, and extremely poor. The 3DE analysis for LV and LA volume measurements was performed with the use of a novel 3DE software (4D LV analysis version 3, LA, TomTec Imaging Systems Unterschleißheim, Germany) by an experienced investigator. Initially, the LV endocardial border was semiautomatically determined after two‐point clicks of the LV apex and center of the mitral valve annulus on the apical four‐chamber, two‐chamber, and long‐axis views extracted from 3DE datasets. The manual adjustment of the endocardial border was performed, when required. Subsequently, the software performed a 3DE speckle tracking analysis throughout one cardiac cycle (Figure 1a). To measure LV mass, epicardial contour was generated on ventricular end‐diastolic three apical views, and the contour was adjusted with manual editing (Figure 1b). The software generated time domain LV volume curves, from which LV volumes and LVEF were calculated (Figure 1c).

For the LA view adjustment, the center of the mitral valve and the LA roof was determined on apical four‐chamber and long‐axis views (Figure 1d). After clicking the tab named “LA tracking revision,” the software automatically determined the LA endocardial border on the three apical long‐axis views. The LA endocardial border could be manually adjusted at the ventricular end‐diastolic and ventricular end‐systolic frames (Figure 1e). The software created 3D cast of the LA wall, and performed a 3DE speckle tracking analysis of the initial LA surface using dynamic LA surface model over time to generate time domain LA volume curves throughout one cardiac cycle, from which LA volumes at ventricular end‐diastole, at ventricular end‐systole, at pre‐A wave, and LA ejection fraction (LAEF) were calculated. Both LV and LA volume curves were simultaneously displayed on the graph (Figure 1f). The LV mass–volume ratio was calculated as LV mass divided by the LV end‐diastolic volume (LVEDV). A guideline recommends that chamber measurements should be reported indexed body surface area (BSA) to allow comparison among individuals in different body size (Lang et al., 2015), indexed LV and LA volumes were calculated (left ventricular end‐diastolic volume index, LVEDVI; left ventricular end‐systolic volume index, LVESVI; left ventricular stroke volume index, LVSVI; maximal LA volume index, LAVImax; minimal LA volume index, LAVImin; LA volume index at pre‐A wave, LAVIprea).

### 2.4 Generation of LV and LA volume ratio curve

Both LV and LA volume curve data were exported as a text file. Each curve was interpolated by 100 points during ventricular systole and 100 points during ventricular diastole from one cardiac cycle using interpolation software (Wordperfect Office X9, Corel Cooperation, Ottawa, Canada). Temporal LVLAVR was calculated on 200 sampling points, and finally, LVLAVR curve was generated in each subject. The mean value of LVLAVR from different age groups were separately plotted on 200 points by male and female subjects.

### 2.5 Doppler analysis

The transmitral peak velocities during early (E) and late (A) ventricular diastole were measured on pulsed‐wave Doppler echocardiography. Mitral annular velocities during early ventricular diastole (e’) at both septal and lateral corners of the mitral annulus were measured, and average E/e’ was obtained in each subject.

### 2.6 Measurement reliability

Intraobserver variability was assessed by having the observer to repeat the measurement of LVEDV, LV end‐systolic volume (LVESV), maximal LA volume (LAVmax), minimal LA volume (LAVmin), LVLAVR at ventricular end‐diastole and ventricular end‐systole 2 weeks apart in 25 randomly selected subjects. Interobserver reliability was determined by employing a second observer to perform these measurements in the same 25 subjects. The intra and interobserver variability was calculated as the absolute differences between the corresponding two measurements in percentages of their mean and intraclass correlation (ICC).

### 2.7 Statistical analysis

Continuous data were presented as mean ± standard deviation (*SD*) or median and interquartile interval (25th–75th percentile) based on the data distribution. Categorical data were expressed as numbers or percentages. The subjects were grouped into age decades (from the 3rd decade to ≥7th decade) based on sex. Group comparison between age decades was conducted using the one‐way analysis of variance (ANOVA). Two‐way ANOVA was performed to determine the effect of age and sex group for each echocardiography parameter. Univariable regression analysis was performed to determine the contribution of anthropometric and echocardiographic parameters for LVLAVR at ventricular end‐diastole and LVLAVR at ventricular end‐systole. Variables showing *p* < .1 were used for multivariable regression analysis with stepwise selection based on Akaike's information criterion. A two‐sided *p* < .05 was considered statistically significant. All statistical analyses were performed using commercial software (SPSS version 24, Chicago, IL; R version 3.4.3, The R Foundation for Statistical Computing, Vienna, Austria).

## 3 RESULTS

Among 313 subjects, the image quality was good in 42 (14%), fair in 131 (41%), poor in 112 (37%), and extremely poor in 26 subjects (8%), respectively. Both LV and LA volume curves could be generated in 280 subjects (Feasibility; 89%). The median frame rate of 3DE datasets was 20/s (25th–75th percentile; 18–26/s). The mean values of LVLAVR at ventricular end‐diastole and at ventricular end‐systole were 5.48 ± 1.53 and 1.25 ± 0.34, respectively, indicating that the LV volume was more than 5.5 times larger than the LA volume at ventricular end‐diastole, and the LV volume was 1.3 times larger than the LA volume at ventricular end‐systole.

### 3.1 Reference values of LV and LA volume ratio

**Figure 2:**
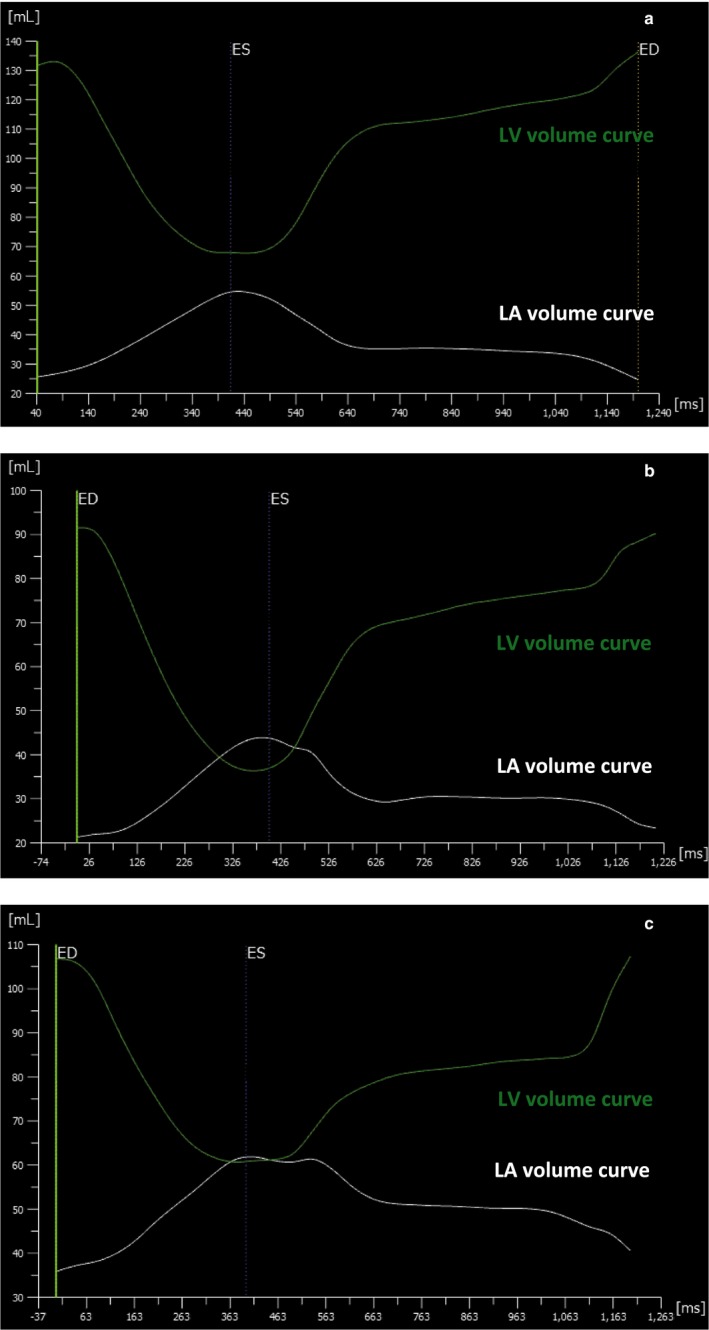
LV and LA volume curves in representative cases. (a) 30‐year‐old male subject. (b) 48‐year‐old female subject. (c) 80‐year‐old male subject

**Figure 3:**
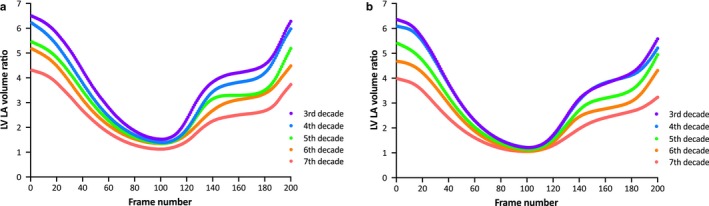
The LVLAVR curves according to groups stratified by age decade. Curves are displayed separately by sex (a, male subjects; b, female subjects)

The reference values for LV and LA volumes, ejection fraction, LV mass, and LVLAVR at ventricular end‐diastole and ventricular end‐systole are summarized according to age group, and displayed separately by male and female subjects (Tables 1 and 2). Among male subjects, LVEDVI, LVESVI, and LVSVI decreased significantly according to advanced aging. In contrast, all LA volume indices at three distinct cardiac phases increased significantly according to age with a simultaneous reduction of LAEF. Thus, the LVLAVR at ventricular end‐diastole significantly reduced according to advancing age, characterizing the highest and lowest values observed in the 3rd (6.80) and ≥7th decades (3.97), respectively. Although less pronounced, the same tendency was also observed in the LVLAVR at ventricular end‐systole. Specifically, the LVLAVR at ventricular end‐systole was the highest in 3rd (1.54), and lowest in ≥7th decades (1.09), respectively. LVLAVR at ventricular end‐diastole had a highest F ratio compared with other left chamber volumetric parameters, reflecting that group means are spread out furthest from the overall mean. The same trend was also observed in female subjects, except that the LVLAVR at ventricular end‐systole showed no age dependency. Figure 2 shows the representative LV and LA volume curves from three subjects with different ages. Figure 3 shows the mean LVLAVR curves stratified by age groups displayed separately by sex.

**Table 1:** Echocardiographic variables according to age decades in male subjects

Variable	All (*n* = 160)	3rd (*n* = 35)	4th (*n* = 46)	5th (*n* = 34)	6th (*n* = 21)	7th (*n* = 24)	*F* ratio	*p* value
BSA (/m^2^)	1.77 ± 0.13	1.76 ± 0.13	1.79 ± 0.12	1.82 ± 0.10	1.76 ± 0.17	1.71 ± 0.12	3.083	.0178
HR (bpm)	63 ± 10	61 ± 12	65 ± 9	64 ± 11	64 ± 9	60 ± 7	1.701	.1505
SBP (mmHg)	129 ± 10	131 ± 9	126 ± 11	130 ± 9	128 ± 12	133 ± 9	2.430	.0500
DBP (mmHg)	76 ± 9	73 ± 9	74 ± 10	77 ± 7	78 ± 7	79 ± 5	3.502	.0091
LVEDVI (ml/m^2^)	74 ± 12	81 ± 12	72 ± 11	75 ± 14	71 ± 11	68 ± 12	4.219	.0029
LVESVI (ml/m^2^)	35 ± 7	38 ± 7	33 ± 6	35 ± 8	34 ± 5	32 ± 6	2.925	.0232
LVSVI (ml/m^2^)	39 ± 8	43 ± 8	39 ± 8	39 ± 7	37 ± 8	36 ± ± 8	3.185	.0154
LVEF (%)	53 ± 6	54 ± 6	54 ± 6	53 ± 4	51 ± 5	53 ± 6	0.643	.6325
LV mass index (g/m^2^)	62 ± 10	64 ± 11	60 ± 10	61 ± 9	64 ± 11	63 ± 9	1.106	.3562
LV mass volume ratio	0.85 ± 0.13	0.80 ± 0.13	0.84 ± 0.13	0.83 ± 0.12	0.91 ± 0.11	0.94 ± 0.14	5.332	.0005
LAVImax (ml/m^2^)	26 ± 6	25 ± 5	25 ± 4	27 ± 5	26 ± 6	30 ± 8	4.069	.0038
LAVIprea (ml/m^2^)	19 ± 5	17 ± 4	16 ± 4	19 ± 4	19 ± 5	24 ± 5	11.069	<.0001
LAVImin (ml/m^2^)	14 ± 4	12 ± 3	12 ± 3	14 ± 3	14 ± 4	18 ± 6	12.229	<.0001
LASVI (ml/m^2^)	13 ± 3	13 ± 3	13 ± 3	13 ± 3	12 ± 4	12 ± 5	0.403	.8064
LAEF (%)	48 ± 9	51 ± 8	52 ± 7	47 ± 7	45 ± 10	40 ± 10	9.422	<.0001
E wave velocity (cm/s)	71 ± 14	76 ± 14	71 ± 16	70 ± 9	66 ± 13	67 ± 16	2.269	.0646
A wave velocity (cm/s)	50 ± 14	40 ± 9	44 ± 9	49 ± 14	54 ± 8	67 ± 14	24.641	<.0001
E/A	1.5 ± 0.5	2.0 ± 0.6	1.7 ± 0.4	1.5 ± 0.3	1.2 ± 0.3	1.0 ± 0.3	21.670	<.0001
e′ (cm/s)	11.2 ± 2.9	13.6 ± 2.3	12.7 ± 2.1	10.9 ± 1.7	9.2 ± 2.2	7.8 ± 1.8	40.132	<.0001
E/e′	6.6 ± 1.9	5.7 ± 1.2	5.6 ± 1.2	6.5 ± 1.3	7.4 ± 1.6	9.0 ± 2.5	22.175	<.0001
LVLAVR at end‐diastole	5.74 ± 1.54	6.80 ± 1.54	6.36 ± 1.31	5.42 ± 1.00	5.23 ± 1.37	3.97 ± 0.85	19.719	<.0001
LVLAVR at end‐systole	1.37 ± 0.35	1.54 ± 0.42	1.39 ± 0.29	1.36 ± 0.31	1.35 ± 0.32	1.09 ± 0.27	5.842	.0002

Abbreviations: BSA, body surface area; DBP, diastolic blood pressure; HR, heart rate; LA, left atrial; LAEF, left atrial ejection fraction; LASVI, left atrial stroke volume index; LAVImax, maximal left atrial volume index; LAVImin, minimal left atrial volume index; LAVIprea, left atrial volume index at pre‐A wave; LV, left ventricular; LVEDVI, left ventricular end‐diastolic volume index; LVEF, left ventricular ejection fraction; LVESVI, left ventricular end‐systolic volume index; LVLAVR, left ventricular and left atrial volume ratio; LVSVI, left ventricular stroke volume index.

**Table 2:** Echocardiographic variables according to age decades in female subjects

Variable	All (*n* = 153)	3rd (*n* = 31)	4th (*n* = 23)	5th (*n* = 30)	6th (*n* = 22)	7th (*n* = 47)	*F* ratio	*p* value
BSA (/m^2^)	1.51 ± 0.11	1.49 ± 0.11	1.52 ± 0.12	1.51 ± 0.12	1.55 ± 0.13	1.49 ± 0.10	1.301	.2723
HR (bpm)	65 ± 9	68 ± 10	65 ± 8	67 ± 10	60 ± 8	63 ± 9	3.126	.0167
SBP (mmHg)	124 ± 11	121 ± 10	121 ± 9	119 ± 10	128 ± 9	129 ± 13	5.846	.0002
DBP (mmHg)	72 ± 9	70 ± 9	72 ± 10	71 ± 9	72 ± 7	74 ± 9	0.908	.4609
LVEDVI (ml/m^2^)	64 ± 10	69 ± 11	68 ± 10	62 ± 8	60 ± 9	63 ± 9	4.288	.0027
LVESVI (ml/m^2^)	28 ± 6	29 ± 6	29 ± 6	26 ± 5	26 ± 7	28 ± 6	1.455	.2197
LVSVI (ml/m^2^)	37 ± 7	40 ± 7	39 ± 7	36 ± 6	33 ± 7	36 ± 6	4.123	.0035
LVEF (%)	57 ± 7	58 ± 6	58 ± 6	58 ± 7	56 ± 8	56 ± 7	0.717	.5815
LV mass index (g/m^2^)	58 ± 10	59 ± 12	59 ± 12	57 ± 9	53 ± 10	61 ± 9	2.018	.0956
LV mass volume ratio	0.91 ± 0.13	0.86 ± 0.13	0.88 ± 0.09	0.92 ± 0.13	0.90 ± 0.16	0.97 ± 0.12	3.903	.0050
LAVImax (ml/m^2^)	25 ± 5	24 ± 5	24 ± 5	24 ± 5	25 ± 5	27 ± 5	1.731	.1470
LAVIprea (ml/m^2^)	18 ± 4	16 ± 3	16 ± 3	16 ± 4	18 ± 4	21 ± 5	12.402	<.0001
LAVImin (ml/m^2^)	13 ± 4	11 ± 2	11 ± 2	12 ± 3	13 ± 4	16 ± 4	16.565	<.0001
LASVI (ml/m^2^)	12 ± 4	14 ± 4	13 ± 4	12 ± 4	12 ± 3	11 ± 3	2.656	.0358
LAEF (%)	48 ± 11	55 ± 7	53 ± 8	49 ± 12	48 ± 9	40 ± 9	13.771	<.0001
E wave velocity (cm/s)	81 ± 17	87 ± 16	91 ± 16	85 ± 14	79 ± 13	71 ± 17	9.085	<.0001
A wave velocity (cm/s)	55 ± 18	44 ± 12	45 ± 13	50 ± 14	55 ± 10	71 ± 17	24.661	<.0001
E/A	1.7 ± 0.7	2.2 ± 0.8	2.2 ± 0.7	1.8 ± 0.5	1.5 ± 0.4	1.0 ± 0.3	26.101	<.0001
e′ (cm/s)	11.1 ± 3.1	14.5 ± 2.0	13.1 ± 1.4	11.9 ± 1.9	10.2 ± 1.4	7.8 ± 1.6	89.421	<.0001
E/e′	7.7 ± 2.0	6.1 ± 1.5	7.1 ± 1.5	7.2 ± 1.2	7.9 ± 1.4	9.2 ± 2.2	17.956	<.0001
LVLAVR at end‐diastole	5.20 ± 1.47	6.51 ± 1.36	6.20 ± 1.26	5.43 ± 1.28	4.67 ± 0.96	4.04 ± 0.81	25.469	<.0001
LVLAVR at end‐systole	1.13 ± 0.29	1.22 ± 0.22	1.22 ± 0.32	1.14 ± 0.33	1.06 ± 0.29	1.06 ± 0.25	2.080	.0870

Abbreviations: BSA, body surface area; DBP, diastolic blood pressure; HR, heart rate; LA, left atrial; LAEF, left atrial ejection fraction; LASVI, left atrial stroke volume index; LAVImax, maximal left atrial volume index; LAVImin, minimal left atrial volume index; LAVIprea, left atrial volume index at pre‐A wave; LV, left ventricular; LVEDVI, left ventricular end‐diastolic volume index; LVEF, left ventricular ejection fraction; LVESVI, left ventricular end‐systolic volume index; LVLAVR, left ventricular and left atrial volume ratio; LVSVI, left ventricular stroke volume index.

### 3.2 Age and sex dependency on the LV and LA volume ratio at ventricular end‐diastole and ventricular end‐systole

Two‐way ANOVA showed that age group was significantly different for LVLAVR at ventricular end‐diastole, but sex group was not different. With regard to the LVLAVR at ventricular end‐systole, both age and sex groups were significantly different.

### 3.3 Contribution of anthropometric and echocardiography parameters

Table 3 describes the contribution of each parameter for LVLAVR at ventricular end‐diastole and ventricular end‐systole. Univariable analysis revealed that age was negatively associated with both LVLAVR at ventricular end‐diastole and ventricular end‐systole. In contrast, the heart rate (HR) and male sex positively correlated with both volume ratios. With regard to LVLAVR at ventricular end‐diastole, LV mass, LAEF, E wave velocity, and E/A were positively associated. E/e’ was negatively associated. However, LVEF had no association. LV mass was positively and LV functional parameters were negatively associated with LVLAVR at ventricular end‐systole. No association was noted between LAEF and LVLAVR at ventricular end‐systole. However, E wave velocity and E/e’ were negatively correlated.

**Table 3:** Contribution of each variable to LV and LA volume ratio

	LVLAVR at end‐diastole				LVLAVR at end‐systole			
	Univariable analysis		Multivariable analysis		Univariable analysis		Multivariable analysis	
	*t* value	*p*	*t* value	*p*	*t* value	*p*	*t* value	*p*
Age	−14.5	<.0001	−9.29	<0.0001	−6.03	<.0001	−8.56	<.0001
Sex (male)	2.96	.0034	2.33	0.0203	6.09	<.0001	2.04	.0420
BSA	1.81	.0711	−3.08	0.0022	3.75	.0002	−3.15	.0018
HR	3.24	.0013	3.48	0.0005	2.82	.0051	2.42	.0161
SBP	−1.74	.0826			1.89	.0592	1.68	.0941
DBP	−2.23	.0265			2.70	.0074		
LVEDV					6.57	<.0001		
LVESV	4.35	<.0001						
LVEF	0.25	.8047			−12.83	<.0001	−13.96	<.0001
LV mass	3.04	.0026	4.33	<0.0001	3.48	.0006	3.52	.0005
LV mass volume ratio	−3.61	.0004			−4.39	<.0001		
LAVmax	−5.86	<.0001						
LAVprea	−11.16	<0.0001			−5.31	<.0001		
LAVmin					−4.31	<.0001		
LAEF	14.49	<.0001	10.36	<0.0001	−1.15	.2521		
E	3.79	.0002			−4.12	<.0001		
E/A	7.58	<.0001			0.58	.5638		
E/e’	−7.30	<.0001			−5.69	<.0001		

Abbreviations: BSA, body surface area; DBP, diastolic blood pressure; HR, heart rate; LA, left atrial; LAEF, left atrial ejection fraction; LAVmax, maximal left atrial volume; LAVmin, minimal left atrial volume; LAVprea, left atrial volume at pre‐A wave; LV, left ventricular; LVEF, left ventricular ejection fraction; LVEDV, left ventricular end‐diastolic volume; LVESV, left ventricular end‐systolic volume; LVLAVR, left ventricular and left atrial volume ratio.

Multivariable analysis including age, sex, BSA, HR, systolic blood pressure (SBP), diastolic blood pressure (DBP), LV mass, and LAEF, showed that age, sex, BSA, HR, LV mass, and LAEF significantly contributed to the LVLAVR at ventricular end‐diastole (adjusted R^2^; 0.615, *p* < .001). Multivariable analysis, including age, sex, BSA, HR, SBP, DBP, LV mass, LVEF, and E/e’, showed that age, sex, BSA, HR, LV mass, and LVEF significantly contributed to the LVLAVR at ventricular end‐systole (adjusted R^2^; 0.550, *p* < .001).

### 3.4 Reliability of 3DE measurements

The intraobserver variability values and ICCs for LVEDV and LVESV, LAVmax, LAVmin, and LVLAVR at ventricular end‐diastole and ventricular end‐systole were 5.7%, 10.0%, 8.3%, 6.8%, 7.3%, and 10.9% and 0.96, 0.91, 0.87, 0.92, 0.94, and 0.91, respectively. The corresponding interobserver variability values and ICCs were 7.6%, 12.5%, 7.9%, 10.4%, 12.6%, and 12.2% and 0.95, 0.90, 0.91, 0.82, 0.80, and 0.89, respectively.

## 4 DISCUSSION

The main findings of this study are summarized as follows: (a) We established the reference values of LVLAVR at ventricular end‐diastole and at ventricular end‐systole; (b) significant age dependency of LVLAVR was observed in both sexes, showing that highest and lowest values were observed in the 3rd and ≥7th decades, respectively; (c) there was also gender dependency, reflecting lower values of LVLAVR at both ventricular end‐diastole and ventricular end‐systole in female subjects than in male subjects; (d) the contribution of echocardiography parameters was different for LVLAVR at ventricular end‐diastole and LVLAVR at ventricular end‐systole, characterizing that LV mass and LA functional parameters were correlated with LVLAVR at ventricular end‐diastole, and LV mass and LV functional parameters were associated with LVLAVR at ventricular end‐systole.

3DE provides accurate and reliable cardiac chamber volumes and their function in clinical practice (Badano et al., 2016; Muraru et al., 2013; Wu et al., 2013). It is generally accepted that aging significantly affects both LV and LA function (Fukuda et al., 2012; Kaku et al., 2011). In this study, LV volumes decrease with age, whereas LVEF remains unchanged, which aligns with previous studies (Fukuda et al., 2012; Kaku et al., 2011). In contrast, LA volumes increase with age. The LVLAVR curves were distinctly different according to age decades by both sexes in this study. The observed differences in LVLAVR among different age decades were most remarkable at ventricular end‐diastole, indicating that the ratio of LVEDV and LAVmin is the most sensitive parameter to reflect age‐related change in both LV and LA volumes. To the best of our knowledge, only one study described the LVLAVR using other imaging modalities. Germans et al. measured LV and LA volumes using manual tracing on endocardial border in every phase of the cardiac cycle with cardiac magnetic resonance steady‐state free precession images in healthy volunteers (Germans et al., 2007). They reported that ratios of LA and LV volumes (this is a reversed ratio compared with the ratio in this study) in ventricular end‐diastole (0.3 vs. 0.2) and ventricular end‐systole (1.6 vs. 1.2) were significantly larger in the older group (*n* = 19; mean age, 51 years) than younger group (*n* = 19; mean age, 29 years), which was in agreement with the results in this study. We expand their findings further using a different imaging modality, 3DE, that is more widely available and semiautomated software that is more user friendly and less time consuming and determined age‐ and sex‐related reference values of LVLAVR in a large number of healthy subjects.

Left atrial functional parameters were closely associated with LVLAVR at ventricular end‐diastole. In contrast, LV functional parameters were associated with LVLAVR at ventricular end‐systole. This reflects that the contribution of LV and LA functional parameters was different at different time points of LVLAVR throughout one cardiac cycle. It is interesting to note that LV mass was associated with LVLAVR at both ventricular end‐diastole and ventricular end‐systole. The LV mass–volume ratio showed stepwise increase according to advancing age in both sexes. As expected, the LV mass–volume ratio had a significant negative association of LVLAVR at both ventricular end‐diastole and ventricular end‐systole. The increased LV mass–volume ratio results in the reduction of LV compliance, resulting in compensated LA volume dilatation to prevent an increase in LA pressure (Germans et al., 2007).

### 4.1 Clinical implications

Because LVLAVR incorporates both LV and LA geometric information, the value reflects the combination of LV and LA function. LVLAVR could detect early abnormalities of LV and LA geometry in patients with systemic disease such as hypertension and diabetes. It may be also useful to elucidate abnormal LV–LA coupling in patients with heart failure with preserved LVEF. Abnormal LVLAVR observed in asymptomatic patients with severe aortic stenosis or mitral regurgitation may provide a clue to perform timely surgical interventions. Detailed assessment of LVLAVR curves clarifies the temporal status of LV–LA coupling at different time points of the cardiac cycle, and thus, would provide further insights for LV–LA coupling. Simultaneous assessment of both LV and LA volume curves also allows the investigation of the total left heart chamber volume and conduit volume, which is another fruitful field for further research (Bowman & Kovács, 2004; Otani, Takeuchi, Lang, & Otsuji, 2010). Further studies would be clarified clinical usefulness of LVLAVR in various cardiovascular diseases.

### 4.2 Study limitations

Several limitations should be acknowledged in this study. First, there was no reference standard to validate the accuracy and reliability of both LV and LA volume measurements. However, we validated the accuracy of LV volume and mass measurements using a previous version of the software, which had no capability for measuring LA volumes (Mizukoshi et al., 2016). Second, the measurements required some manual editing that produced measurement variabilities. Although the use of 3DE fully automated left chamber quantification software eliminates this limitation, it requires specific mode of 3DE acquisition in a specific ultrasound manufacturer. Finally, further multination observational studies should be required to determine whether racial differences exist in the reference range of the LVLAVR.

## 5 CONCLUSIONS

We determined age‐ and sex‐specific reference values of LVLAVR that could be useful to identify subtle change in both LV and LA geometry and function in various clinical scenarios.

## CONFLICT OF INTEREST

Takeuchi has received equipment grant and speaker's honoraria from TomTec Imaging Systems (Unterschleißheim, Germany). The other authors report no conflicts.
